# Pilot trial protocol: community intervention to improve depressive symptoms among Peruvian older adults

**DOI:** 10.1186/s40814-024-01540-1

**Published:** 2024-08-22

**Authors:** Tatiana Cruz-Riquelme, Alejandro Zevallos-Morales, Ivonne Carrión, Diego Otero-Oyague, Vanessa Patiño, Dafne Lastra, Rubén Valle, José F. Parodi, Suzanne L. Pollard, Lesley Steinman, Joseph J. Gallo, Oscar Flores-Flores

**Affiliations:** 1 Facultad de Medicina Humana, Centro de Investigación del Envejecimiento (CIEN), Universidad de San Martin de Porres, Lima, Peru; 2 Asociación Benéfica PRISMA, Lima, Peru; 3 Facultad de Medicina Humana, Centro de Investigación en Epidemiología Clínica y Medicina Basada en Evidencias, Universidad de San Martin de Porres, Lima, Peru; 4 Center for Global Non-Communicable Disease Research and Training, School of Medicine, Johns Hopkins University, Baltimore, MD USA; 5 Health Promotion Research Center, Department of Health Systems and Population Health, University of Washington School of Public Health, Seattle, WA USA; 6 Department of Mental Health, Johns Hopkins Bloomberg School of Public Health, Baltimore, MD USA; 7 Department of General Internal Medicine, School of Medicine, Johns Hopkins University, Baltimore, MD USA

**Keywords:** Depression, Anxiety, Old age, Feasibility studies, Implementation, Adaptations, Community health workers, Loneliness

## Abstract

**Background:**

Non-pharmacological interventions have proven effective at alleviating depression and anxiety symptoms in older adults. Methodological refinement and testing of these interventions in new contexts are needed on a small scale before their effectiveness and implementation can be evaluated. The purpose of this pilot study is to assess the feasibility of a future large-scale trial comparing an adapted mental health multi-component evidence-based intervention (VIDACTIVA) versus standard care for older adults experiencing depression symptoms in urban, resource-limited settings in Lima, Peru. Furthermore, this study will explore the acceptability, feasibility, and fidelity of implementing the intervention.

**Methods:**

We will conduct an open-label, mixed methods pilot feasibility study with two parallel groups. A total of 64 older adults, stratified by sex, will be randomized at a 1:1 ratio to either the “intervention” or “control.” Participants will be followed for 22 weeks after enrollment. Those in the intervention group will receive eight VIDACTIVA sessions administered by community health workers (CHWs) over 14 weeks, with an additional eight weeks of follow-up. Participants in the control group will receive two psychoeducation sessions from a study fieldworker and will be directed to health care centers. Standard care does not involve CHWs. We will evaluate screening rates, recruitment strategies, retention rates, the acceptability of randomization, and assessments. Additionally, we will assess preliminary implementation outcomes—acceptability, feasibility, and fidelity—from the perspectives of CHWs (interventionists), older adults (main participants), older adults’ relatives, and healthcare professionals.

**Discussion:**

If the findings from this feasibility trial are favorable, a fully powered randomized controlled trial will be conducted to evaluate `both the effectiveness and implementation of the intervention. This research will make a substantial contribution to the field of mental health in older adults, particularly by emphasizing a meticulous examination and documentation of the implementation process. By doing so, this study will offer valuable methodologies and metrics for adapting and assessing mental health interventions tailored to the unique needs of older adults in resource-constrained contexts and diverse cultural settings.

**Trial registration:**

The current trial registration number is NCT06065020, which was registered on 26th September 2023.

## Background

Depression is highly prevalent among older adults [1] and affects approximately one in three older adults worldwide [2]. Depression often coexists with anxiety symptoms [3] and are linked to adverse outcomes such as falls, disability, cognitive impairment, dementia, suboptimal management of chronic conditions, and an increased risk of suicide [4–6]. Loneliness frequently accompanies depression [7] and is independently associated with negative health consequences, including mortality [8].

Despite the availability of effective non-pharmacological treatments such as behavioral activation and problem-solving therapy for late-life depression and anxiety symptoms [6, 9, 10], these therapies often remain out of reach for those in need. The scarcity of mental health professionals, including psychologists and psychiatrists, is a significant barrier, particularly in low- and middle-income countries (LMICs) [11, 12]. To address this challenge, the World Health Organization (WHO) promotes task-shifting and task-sharing interventions that integrate non-specialists into routine healthcare delivery [13]. Task-shifting involves redistributing tasks from highly trained professionals to individuals with less extensive training, including non-professionals to maximize resource efficiency. Task-sharing underscores the importance of collaborative care within a team of providers, with mental health specialists transitioning from direct service providers to trainers, supervisors, and consultants [14]. Task-sharing interventions that tap into community resources, such as community health workers (CHWs), not only expand the workforce but also reduce mental health stigma and can provide essential support to older adults [15, 16].

In Peru, despite its classification as an upper-middle-income country, a significant gap exists between the demand for and supply of mental health services, with historical limitations in centralized care in psychiatric hospitals. However, in the last 12 years, there has been an increase in political will highlighted by the approval of Law 29889, which guarantees rights and promotes comprehensive mental health reform. This included the creation of community mental health centers (CMHCs) to provide specialized mental health care to communities, strengthening primary care [17–19]. Typically, CMHCs are staffed with specialized mental health professionals, including psychiatrists and psychologists, while the primary care centers may occasionally have psychologists on their teams. CMHCs offer care for moderate and severe cases while providing technical support to primary care centers [18].

Among Peruvian older adults with depression, more than 75% do not receive any form of treatment [20]. Psychiatrist-to-population ratio is 3 per 100,000 people, and the psychologist-to-population ratio is 10 per 100,000 [21]. Given these limited structural and human resources for mental health, there is an urgent need for innovative approaches to delivering mental health care to older adults.

We present a protocol for a pilot feasibility trial of a community-based mental health multi-component intervention designed to alleviate depressive symptoms among Peruvian older adults living in urban, resource-limited settings. This trial builds upon previous community-level studies that enhanced our understanding of older adults’ perceptions of mental health and their specific needs in the Peruvian context [22, 23]. In alignment with the community-based approach endorsed by the Peruvian Ministry of Health and the WHO, we iteratively adapted components of the Program to Encourage Active Rewarding Lives (PEARLS), an evidence-based intervention for underserved older adults with depression developed in the USA [24, 25], to create the *Vidas Activas y Valiosas *(*VIDACTIVA*) program tailored to the Peruvian context. The primary components of VIDACTIVA include problem solving [10], behavioral activation [26], and psychoeducation provided by CHWs under the supervision of a clinical team.

The objective of this protocol is to evaluate the feasibility of conducting a larger-scale clinical trial while simultaneously assessing preliminary implementation and clinical outcomes. We will assess the acceptability and feasibility of the intervention from the various perspectives of those involved (CHWs, older adults, family members, and health professionals) and evaluate the fidelity of intervention delivery by CHWs within a resource-constrained urban setting.

## Methods

### Study setting

Our study will be conducted in Villa El Salvador (VES), an urban district situated to the south of Lima. VES is home to a population of 432,170 residents, among whom 7% are aged 60 years and older [27]. The majority of older adults in this area are Andean migrants who initially settled here during 1970s. As of 2022, the poverty rate among older adults in this district was high, reaching 28%, with an estimated monthly income averaging less than 358.60 Nuevos Soles (approximately 95 US dollars) [28]. The life expectancy in VES is slightly below the national average, at 72.8 years, compared to the country’s average of 76.9 years. Within the older adult population, 20% of respondents reported experiencing illiteracy, and half lacked access to a pension [29]. In VES, in 2023, there were three CMHCs and 16 primary health centers serving the community. Both primary health centers and CMHCs have CHWs, who are community members that volunteer to support various health activities. The size of CHW networks and their specific duties and responsibilities can vary significantly depending on the health center.

### Study design and sample size

**Fig. 1:**
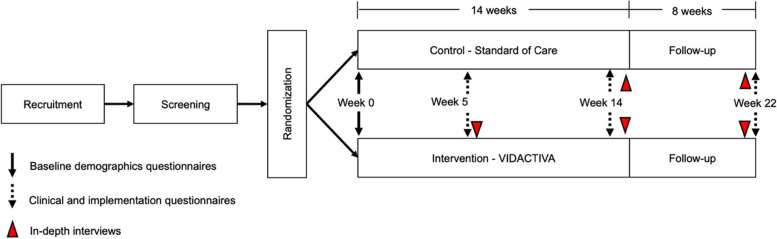
Schematic of the protocol study design and assessments

Our study adopts a mixed-method feasibility pilot trial, employing an open-label design with a type 2 implementation-effectiveness focus and featuring two parallel groups. We plan to randomize a total of 64 older adults (60 years and older) stratified by sex and dividing them equally into “control” and “intervention” groups. The decision to include 64 participants was influenced by the availability of pairs of trained CHWs, who will be responsible for delivering the intervention to a group of 32 participants each. The trial is scheduled to run for a duration of 22 weeks from the start of enrollment. For a visual representation of our study design, please refer to Fig. 1.

### Study participants

Our study encompasses four distinct participant categories:

#### Older adults

This group will be the focus of our intervention, and they will be randomized into either the intervention or control group. Older adults will have to meet the following inclusion criteria:

- Older adults ≥ 60 years old with the capacity to provide consent.
- Current residence in VES, Lima, Perú.
- Depressive symptoms, determined by a score equal to or greater than 6 on the Patient Health Questionnaire-9 (PHQ-9).

The exclusion criteria for will be:

- Previous self-reported diagnosis of bipolar disorder, dissociative disorder, psychosis, or dementia.
- Alcohol abuse, using the 4-item CAGE screening questionnaire. Individuals with a score equal or greater than 2 will be excluded [30].
- Cognitive impairment based on six-items screener [31]. The score ranges from 0 (worst) to 6 (best). If participants score less than 3, they would be excluded.

#### Relatives or family members

This category comprises family members or relatives of the older adult participants. Preliminary work has revealed that older adults do not live alone and that family members can play significant roles as both facilitators and/or barriers to the acceptance of mental health care [22, 23]. Their involvement will be on sharing their perspectives regarding any observed changes in the older adult receiving the intervention and identifying strategies for engaging relatives in potential future family-oriented interventions. To be eligible for participation in this category, individuals must meet the following inclusion criteria:

- An adult aged 18 years or older with the capacity to provide informed consent.
- Reside in the same household as an older adult who is participating in the intervention group.
- Be referred by an older adult who is actively participating in the intervention group.

#### Health professionals

This category encompasses a diverse group of health professionals, such as nurses, technicians, social workers, psychologists, and psychiatrists, who are actively engaged in providing care or holding managerial roles within the healthcare system. Their pivotal role in this study involves offering valuable insights, perspectives, and opinions concerning the delivery of the intervention. To be eligible for participation in this category, individuals must meet the following inclusion criteria:

- Be currently employed at primary health care centers or community mental health centers or within the local Ministry of Health administration in the district of VES.
- A minimum of 6 months of work experience in the district of VES.

#### Community health workers

CHWs will play a crucial role in delivering the intervention. Fourteen CHWs will actively participate in this pilot trial. These CHWs have undergone comprehensive training totaling 27 h, carefully designed, and conducted by our clinical team. The training curriculum was centered on the nuances of mental health in older adults and on the specific components of the intervention. Additionally, these CHWs were actively engaged in the iterative adaptation phase preceding this pilot trial. We will obtain and register their experiences, opinions, and insights regarding the intervention delivery.

## Recruitment

### Recruitment and enrollment of older adults

To ensure effective recruitment and enrollment of older adults, our approach will be led by two fieldworkers. We will employ two primary strategies:

- Engagement with community organizations: Initially, we will collaborate with leaders of community organizations in which older adults actively participate, such as Soup Kitchens and Church gatherings. In this collaborative effort, we will provide essential information about our study to these organizations. Subsequently, we will organize group meetings within these community settings to personally acquaint older adults with the study details. We will thoroughly explain the study and leave contact information for our study coordinator, making it convenient for interested individuals to enroll.
- Door-to-Door invitations: Recognizing that some older adults may not actively participate in community groups, we will take a more proactive approach. Our team will visit residences on the outskirts of the VES district, extending personal invitations to potential participants. We believe that reaching out to older adults who may not be part of community organizations will allow us to include those who are potentially most in need.

After providing written informed consent, individuals will undergo a screening process based on our eligibility criteria. For those who express interest but do not meet the eligibility requirements, we will provide valuable information about their nearest primary care center. This approach ensures that even those ineligibles for our study receive guidance on accessing healthcare resources.

### Recruitment of relatives of older adults

Relatives of older adults will be recruited directly through referrals from older adults participating in the trial. We will initiate contact through phone calls to obtain consent for conducting in-depth interviews.

### Recruitment of health professionals

Health professionals, with whom we collaborated during the previous iterative pilot study of adapting the VIDACTIVA, will be invited for in-depth interviews about their perspectives on the intervention. We will reach out to them via phone calls.

### Community health workers

CHWs who have previously worked with us in the iterative pilot will continue their involvement in this trial.

### Randomization

Upon establishing the eligibility of older adults who consented to join the study, they will undergo randomization administered through an online platform (accessible at https://www.sealedenvelope.com/) by the research team. This computer-based randomization system will employ a 1:1 allocation scheme to assign individuals to either the intervention group or the usual care group. The allocation will be stratified based on sex and will utilize random permuted blocks of varying sizes.

### Blinding

Fieldworkers in charge of periodic assessments will be blinded to the treatment allocation. Researchers and study participants will not be blinded to treatment allocation due to the nature of the intervention.

## Study groups

### Intervention arm

**Fig. 2:**
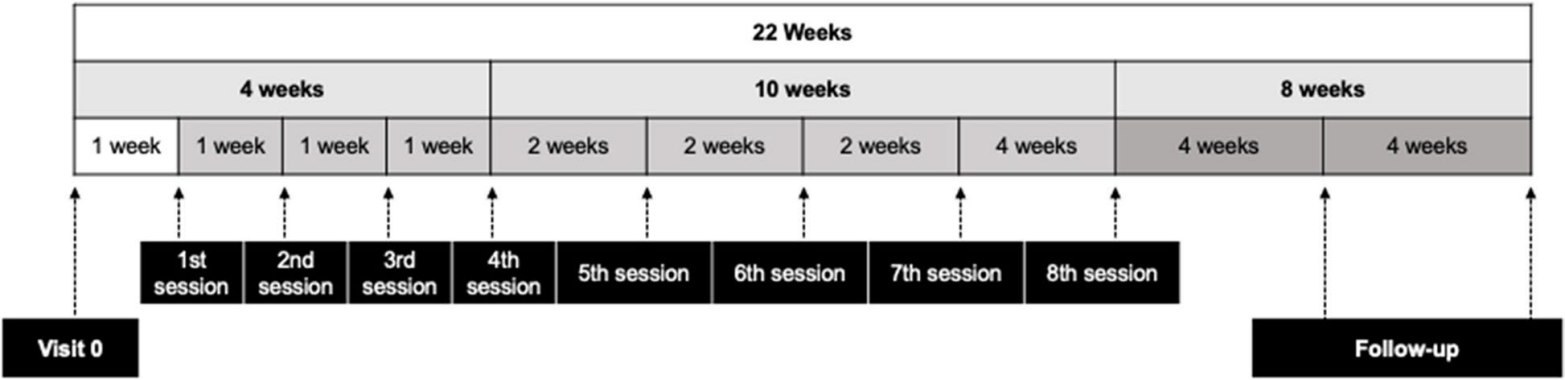
Timeline of the intervention sessions for VIDACTIVA. Visit 0: This initial visit is an informal introduction by CHWs. Sessions 1–8: These structured sessions share the same format but progressively taper. The first 4 sessions occur weekly, session 5th to 7th occur every 2 weeks and session 8th is 4 weeks after 7th session. Follow-up: Two monthly phone calls also conducted by CHWs

In the intervention arm, participants will receive the comprehensive VIDACTIVA intervention, which consists of eight in-person sessions led by two CHWs over a 14-week period. There will be an additional eight-week follow-up phase, during which participants will receive monthly phone calls (a total of two calls) to maintain contact and support (see Fig. 2).

Each pair of CHWs will work closely with one older adult participant throughout the intervention journey. To establish a strong foundation, a “meeting zero” will occur before the first session. During this introductory meeting, CHWs will have the opportunity to introduce themselves, foster mutual understanding, and build trust with the participant.

The core VIDACTIVA intervention consists of eight sessions, each encompassing the following components:

- Problem-solving exercises.
- Planning of pleasant, social, and physical activities.
- Psychoeducation on depression and anxiety in older adults.

For a detailed breakdown of the content covered in each session, please refer to Table 1.

**Table 1:** Components of the VIDACTIVA intervention administered by CHWs

Component	Description
Problem-solving exercises	These structured exercises aim to train older adults in problem-solving activities. During these exercises, older adults take the lead, with the CHW acting as a facilitator. The CHW's role is to guide rather than provide advice. The process involves selecting a problem, setting a goal, brainstorming potential solutions, choosing the best solution, and taking steps toward its achievement
Behavioral activation	This component involves planning and "scheduling" physical, social, and/or enjoyable activities that older adults select based on their interests and capabilities. Its aim is to reduce sedentary behavior and assist in organizing activities. Individuals with depression often withdraw from activities they once enjoyed, so scheduling meaningful activities in advance can help enhance mood and energy levels
*Education on depressive and anxiety symptoms	This component primarily aims to convey and emphasize three key points: • Symptoms such as sadness, lack of energy, and sleep problems may be indicative of depression • Depressive or anxiety symptoms can be connected to unresolved issues • Physical activity, or body activation, can play a role in reducing depressive symptoms Additionally, participants will receive essential information about available community resources for improving mental health, including services, community mental health centers, and senior citizens’ clubs. This information aims to contribute to the reduction of stigma associated with mental health

Participants in the intervention group will receive the same components at each session. *Participants in the control group will receive 2 psychoeducation sessions and referral to primary care centers by a study fieldworker

Throughout the trial, alongside their individual sessions with older adult participants, CHWs will actively engage in weekly group and individual supervision sessions led by a clinical psychologist. These supervision meetings will serve as platforms for discussing challenges and difficulties encountered during the intervention, providing constructive feedback, tracking the progress of each older adult, and offering space for CHWs to share their thoughts and emotions. Importantly, these meetings will also facilitate peer support and emphasize self-care among CHWs.

### Control group

Participants will receive psychoeducation sessions addressing depression and anxiety at older age, which will be delivered through two separate visits (week 0 and week 10) by a trained study fieldworker.

During the initial visit at week 0, participants will receive comprehensive information about the community resources available to them. They will also receive guidance on how to access standard care services provided at their local health canter. If a participant is already receiving some form of mental health care, educational sessions will still be offered, enhancing their existing care. Importantly, any changes in mental health treatment status, whether initiation or cessation, will be meticulously recorded during the study assessments.

### Measures and evaluation

Research evaluations will include baseline (week 0), middle (week 5), end of intervention (week 14), and at the end of follow-up (week 22); see Fig. 1. Quantitative data from questionnaires will be electronically collected by a trained fieldworker using REDCap electronic data capture tools and tablets.

### Baseline demographics

After providing informed consent, baseline information will be obtained on age, sex, gender, literacy, civil status, working status, household composition, health insurance, and whether participants are caregivers. Additionally, we will ask about the medications used and the presence of chronic conditions, namely, hypertension, diabetes, chronic respiratory disease, osteoporosis, cataracts, glaucoma, chronic kidney disease, cancer, anxiety disorders, and mood disorders. Furthermore, we will obtain information about self-reported hearing status [32] and self-reported vision status [33]. Finally, we will measure quality of life with the EuroQoL-5D (EQ-5D-5L) [34], independence (Katz Index) [35], food insecurity scale [36], physical frailty [37], and perceived social support (Medical Outcomes Study Social Support Survey—mMOS-SS) [38]. We will obtain information on whether the participant is receiving any type of mental health therapy (pharmacotherapy or psychotherapy).

### Feasibility of carrying out a larger trial

We will collect feasibility data from the pilot trial, which will allow us to assess and plan a full-scale randomized controlled trial (RCT). A comprehensive overview of these feasibility domains, encompassing quantitative indicators, qualitative data, and predefined success criteria, can be found in Table 2. The qualitative data will be sourced from a variety of avenues, including field notes maintained by the study coordinator and interviews with participants. Special attention will be given to those who chose not to participate or who withdrew from the study. These interviews will allow us to elucidate the underlying reasons and barriers contributing to their decisions, thereby enhancing our understanding of the trial’s feasibility and participant perspectives.

**Table 2:** Feasibility assessment for conducting a fully powered randomized controlled trial

Item	Quantitative assessments	Qualitative interviews	Target/criteria for success
Screening	# participants screened/month	Barriers and facilitators to screening and recruitment	
Enrollment	# participants enrolled/month Average time to reach enrollment goal		80% older adults enrolled/4 months
Retention	% older adults who remain in the study in both study groups	Reasons for dropout	80% trial retention at both groups at week-14 75% trial retention at both groups at week-22
Assessments	% of assessment administered as planned	Barriers and facilitators to delivering the intervention	75% evaluations complete

Our progression criteria are not solely based on quantitative success thresholds; instead, we will integrate qualitative data from semi-structured interviews with the quantitative criteria for each domain described in Table 2. For instance, if we do not reach the screening and recruitment targets, we will explore from the fieldworkers' perspectives how we can improve for a future phase. Reasons could include the number of fieldworkers, the way the study is presented, the recruitment strategies, or other factors. In the domain of retention, if a high proportion of participants drop out after being randomized to the control group, we will use the semi-structured interviews to understand their reasons. This feedback may lead us to reconsider the study design, or the type of control used for the future trial.

### Implementation outcome measures

Implementation outcomes serve as a vital lens through which we can unravel the intricacies of the implementation process, allowing us to pinpoint and address intermediate factors that may impact the success of an intervention within its specific context [39, 40]. These outcomes hold immense significance because they enable us to assess the effectiveness of delivery of VIDACTIVA, paving the way for future research and practice. Without successful implementation, clinical outcomes remain elusive.

To comprehensively evaluate the acceptability, feasibility, and fidelity of the intervention delivery, we will employ mixed methods approaches [37, 38]. A concise overview of the domains, methodologies, and schedule for data collection is provided in Table 3, which offers a clear roadmap for our assessment process.

**Table 3:** Evaluation of implementation outcome domains: definition, method, data source, and timeline

Implementation domain	Definition	Method	Data source	Timeline
Acceptability	Extent to which an intervention aligns with an individual’s preferences, needs, and expectations	In-depth interviews Acceptability Likert Scale	Older adults Relatives of older adults Health professionals	week-5, week-14, week-22 week-14 week-14
		Fieldnotes	Supervision sessions	weekly
Feasibility	Practicality of implementation, considering available resources and circumstances	In-depth interviews Feasibility Likert Scale	Older adults Relatives of older adults Health professionals	week-5, week-14, week-22 week-14 week-14
		Fieldnotes	Supervision sessions	weekly
Fidelity	The degree to which an intervention is delivered as intended	Indicators: % of older adults who receive 4 and 8 sessions % of older adults who schedule and do pleasant, social, and physical activities % of older adults who agree to have in-home sessions vs sessions outside % of sessions delivery according to schedule Average time of study visits	Log Study coordinator	Throughout the trial
		Fieldnotes	Supervision sessions	Weekly
		Audio-recording of visits + Checklist	CHWs-Older Adult interaction	Week-6 (Session 5)

### Acceptability and feasibility

To evaluate the acceptability and feasibility of our intervention, we will employ an explanatory sequential mixed-methods approach. For older adults, we will start by collecting quantitative data through the Acceptability of Intervention Measure (AIM) and Feasibility of Intervention Measure (FIM) Likert scales [41]. Subsequently, we will conduct in-depth interviews with a subset of 16–20 older adults, including those who were both less and more satisfied with the intervention. These interviews aim to delve into the participants’ experiences with the program. During these interviews, we will explore feelings about the intervention, thoughts on the activities facilitated by the CHWs, aspects older adults’ value and find satisfying, as well as areas they believe could be improved.

We will use a convergent mixed-methods approach with CHWs, older adult relatives, and health professionals, combining quantitative scores from the Feasibility and Acceptability Likert scales with in-depth interviews, all within the same phase [42]. This comprehensive approach ensures a holistic interpretation of the data.

Interviews with CHWs will focus on various aspects, including challenges faced while providing the intervention to different older adults, time invested, compensation, self-care practices, and overall satisfaction. In addition to these interviews, we will collect valuable insights during weekly supervision sessions, where a designated note-taker will document CHWs’ experiences and interactions with participants and their families, if applicable.

Interviews with family members will explore their perceptions of mental health care, their acceptance of the intervention, positive changes observed in the family member receiving the intervention, and various other related topics.

Interviews with health professionals will seek to understand their perspectives on the long-term feasibility of the intervention and explore potential avenues for support and collaboration, such as funding opportunities and referrals. This multifaceted approach ensures a thorough examination of the intervention's acceptability and feasibility from multiple perspectives.

### Fidelity

We will evaluate the fidelity of the delivery of intervention using a convergent mixed methods approach that includes quantitative indicators and in-depth interviews. The details are displayed in Table 3.

We will analyze various indicators derived from the PEARLS fidelity instrument [43]. Additionally, we will audio record one session (middle session) to evaluate the interaction and relationships of CHWs with older adults, communication skills, and rapport. We will closely evaluate the execution of problem-solving exercises and the scheduling of activities. To facilitate this assessment, we are developing a concise checklist outlining the essential steps of problem-solving exercises.

This multifaceted approach to assessing fidelity will offer a nuanced perspective on the accuracy and integrity of intervention delivery by CHWs, providing rich data that will help guide how to adapt VIDACTIVA intervention and implementation for future RCTs.

### Clinical measures

The preliminary primary clinical outcome will be depressive symptoms. The secondary outcomes will include anxiety symptoms and loneliness.

#### Depressive symptoms

To assess the severity of depressive symptoms, we will use the self-report Patient Health Questionnaire-9 (PHQ-9). We will utilize the Spanish version validated for use in Peruvian population [44, 45]. The PHQ-9 is a 9-item questionnaire. Each item is rated on a 4-point scale. Scores within the range of 5 to 9 indicate mild symptoms, scores between 10 and 14 signify moderate symptoms, a range of 15 to 19 indicates moderately severe symptoms, and scores of > 20 suggest severe depression.

#### Anxiety symptoms

We will evaluate the severity of anxiety symptoms using the Generalized Anxiety Disorder-7 (GAD-7), a validated Spanish version [46]. The questionnaire has 7 items, each with a 4-point scale, with scores between 0 and 21 points. Scores are consider as follows: 0–4 (minimal), 5–9 (mild), 10–14 (moderate), and 15–21 (severe) symptoms [46]. The GAD-7 has demonstrated excellent psychometric properties [47].

#### Loneliness

Loneliness will be assessed using the Three-Item Loneliness Scale (TIL-3) in its Spanish version [48, 49]. Respondents will indicate how frequently they experience feelings of lacking companionship; being excluded; and experiencing social isolation. Participants will provide responses on a 3-point Likert scale, ranging from 1 “hardly ever” to 3 “often.” Individual responses will be summed, with higher scores indicative of greater loneliness. The TIL-3 yields a score ranging from 3 to 9.

### Data analysis

To visualize participant flow, we will employ a CONSORT diagram [50]. For a comprehensive overview, we will provide summary statistics, including means, standard deviations, medians, interquartile ranges, counts, or percentages, for all baseline characteristics, implementation outcomes, and clinical measures.

To provide a comprehensive view of the intervention’s delivery process, we will adopt a mixed-methods approach [42, 51]. This approach will facilitate the creation of a detailed and holistic portrayal of the intervention's implementation. Our plan includes obtaining meta-inferences and creating joint displays for both the implementation and clinical outcomes.

Qualitative data will be organized using MAXQDA software (VERBI GmbH, Berlin, Version 18.2). Qualitative data from in-depth interviews will be analyzed by a multi-disciplinary team. We will examine the initial transcripts and generated preliminary coding categories through an inductive process using the constant comparative method [52]. Through this analytical process, we will identify patterns and commonalities, as well as particularities, that will enhance our understanding of the acceptability and feasibility of the intervention from the perspective of participants, their relatives, CHWs, and other health professionals involved in providing care to older people.

Clinical outcome measures will be summarized for each trial arm at specific time points. We will estimate effect sizes as between-group mean differences for each outcome using linear mixed models post-trial (week 14) and at the end of the follow-up period (week 22). We will present parameter estimates along with 95% confidence intervals. It is important to note that hypothesis testing will not be conducted because this is a pilot trial. All the statistical analyses will be performed using Stata v.17 software provided by StataCorp (College Station, TX).

### Planned interim analysis and stopping rules

No interim analysis has been planned.

### Multiple testing

Given that this feasibility study is exploratory and that there is no hypothesis testing, there will be no adjustment made to the analysis for multiple testing.

### Ethics

The following protocol study has been approved by Universidad de San Martín de Porres (USMP) Institutional Ethical Review Board (reference #948–2023) and Asociacion Benefica PRISMA Ethical Committee (reference #CE0453.23): This trial is registered at Clinical Trials.gov with the identifier NCT06065020. We will obtain written informed consent from the study participants.

## Discussion

We present the justification and methods for the pilot feasibility trial “VIDACTIVA,” a multi-component CHW-lead intervention for Peruvian older adults experiencing depressive and anxiety symptoms. The VIDACTIVA intervention has undergone iterative adaptation from the well-established “Program to Encourage Active, Rewarding Lives” (PEARLS), which has been successfully implemented in the USA over the past 15 years [24, 25, 53].

While PEARLS has indeed shown significant positive outcomes in addressing depressive symptoms and improving quality of life [54], it is essential to recognize the limitations in generalizing its results to low-resource settings and distinct cultural beyond the United States and in the adoption of CHW-led mental health interventions.

The primary objective of this pilot feasibility trial is to bridge these knowledge gaps and prepare for a more extensive and comprehensive trial. The insights gathered throughout this trial will be instrumental in refining our strategies for the main trial. Pilot feasibility testing becomes notably intricate when conducted with populations that have historically been underrepresented in clinical trials, such as older adults [55], and in low-resource settings characterized by significant organizational, cultural, and infrastructural challenges [42]. Explicit attention to culture is needed to obtain health equity [56]. The findings from this study have the potential to inform future adaptations, assessments, and implementations of interventions involving CHWs in urban resource-constrained settings, thereby expanding the mental health workforce to address pressing needs.

While the intervention primarily targets the alleviation of depressive and anxiety symptoms, we recognize that most older adults experience multiple chronic conditions, a phenomenon known as multimorbidity [57]. We anticipate that case discussions during supervision sessions can provide valuable insights into other potential components of a future or refined CHW-led intervention. Furthermore, a key element of the problem-solving component is that older adults themselves select the problems they wish to address and propose solutions. During these exercises, the identified problem may relate to other chronic conditions that older adults are managing (e.g., improving diabetes control). This user-centered approach has the potential to indirectly impact the management of other health conditions, illustrating the broader positive effects of the intervention. This approach aligns with the efforts to integrate mental health with non-communicable diseases care, centering the experience of the people living with mental illness (PAHO) [16, 58].

### Trial status

At the time of manuscript submission, recruitment for this study was ongoing. Recruitment started on 23rd October 2023, is not yet complete and is due to completion on 30th July 2024. This is protocol version 1.2. The trial sponsor is the Universidad de San Martin de Porres.

## Data Availability

Not applicable.

## Abbreviations

CHW: Community health worker
CMHC: Community mental health center
RCT: Randomized controlled trial
LMICs: Low-middle income countries
USMP: Universidad de San Martin de Porres
PEARLS: Program to Encourage Active, Rewarding Lives
WHO: World Health Organization
PAHO: Pan-American Health Organization

## References

[1] Zenebe Y, Akele B, M WS, Necho M. Prevalence and determinants of depression among old age: a systematic review and meta-analysis. Ann Gen Psychiatry. 2021;20(1):55. 34922595 10.1186/s12991-021-00375-x PMC8684627

[2] Cai H, Jin Y, Liu R, Zhang Q, Su Z, Ungvari GS, et al. Global prevalence of depression in older adults: A systematic review and meta-analysis of epidemiological surveys. Asian J Psychiatr. 2023;80:103417. 36587492 10.1016/j.ajp.2022.103417

[3] Prina AM, Ferri CP, Guerra M, Brayne C, Prince M. Co-occurrence of anxiety and depression amongst older adults in low- and middle-income countries: findings from the 10/66 study. Psychol Med. 2011;41(10):2047–56. 21466747 10.1017/S0033291711000444

[4] Ismail Z, Fischer C, McCall WV. What characterizes late-life depression? Psychiatr Clin North Am. 2013;36(4):483–96. 24229652 10.1016/j.psc.2013.08.010

[5] Kang HJ, Bae KY, Kim SW, Shin IS, Yoon JS, Kim JM. Anxiety symptoms in Korean elderly individuals: a two-year longitudinal community study. Int Psychogeriatr. 2016;28(3):423–33. 26299311 10.1017/S1041610215001301

[6] Frost R, Bauernfreund Y, Walters K. Non-pharmacological interventions for depression/anxiety in older adults with physical comorbidities affecting functioning: systematic review and meta-analysis. Int Psychogeriatr. 2019;31(8):1121–36. 30479241 10.1017/S1041610218001564 PMC6398582

[7] Haroz EE, Ritchey M, Bass JK, Kohrt BA, Augustinavicius J, Michalopoulos L, et al. How is depression experienced around the world? A systematic review of qualitative literature. Soc Sci Med. 2017;183:151–62. 28069271 10.1016/j.socscimed.2016.12.030 PMC5488686

[8] Gao Q, Prina AM, Prince M, Acosta D, Luisa Sosa A, Guerra M, et al. Loneliness among older adults in Latin America, China, and India: prevalence, correlates and association with mortality. Int J Public Health. 2021;66:604449. 34744572 10.3389/ijph.2021.604449 PMC8565277

[9] Holvast F, Massoudi B, Oude Voshaar RC, Verhaak PFM. Non-pharmacological treatment for depressed older patients in primary care: A systematic review and meta-analysis. PLoS ONE. 2017;12(9):e0184666. 28938015 10.1371/journal.pone.0184666 PMC5609744

[10] Bell AC, D’Zurilla TJ. Problem-solving therapy for depression: a meta-analysis. Clin Psychol Rev. 2009;29(4):348–53. 19299058 10.1016/j.cpr.2009.02.003

[11] Shifting WT. Rational redistribution of tasks among health workforce teams: global recommendations and guidelines. Geneva: World Health Organization; 2008.

[12] Kohrt BA, Asher L, Bhardwaj A, Fazel M, Jordans MJD, Mutamba BB, et al. The Role of Communities in Mental Health Care in Low- and Middle-Income Countries: A Meta-Review of Components and Competencies. Int J Environ Res Public Health. 2018;15(6):1279. 29914185 10.3390/ijerph15061279 PMC6025474

[13] Patel V, Saxena S, Lund C, Thornicroft G, Baingana F, Bolton P, et al. The Lancet Commission on global mental health and sustainable development. The Lancet. 2018;392(10157):1553–98. 10.1016/S0140-6736(18)31612-X 30314863

[14] Patel V. Global mental health: from science to action. Harv Rev Psychiatry. 2012;20(1):6–12. 22335178 10.3109/10673229.2012.649108 PMC3335087

[15] Hoeft TJ, Fortney JC, Patel V, Unützer J. Task-sharing approaches to improve mental health care in rural and other low-resource settings: a systematic review. J Rural Health. 2018;34(1):48–62. 28084667 10.1111/jrh.12229 PMC5509535

[16] Patel V, Saxena S, Lund C, Kohrt B, Kieling C, Sunkel C, et al. Transforming mental health systems globally: principles and policy recommendations. The Lancet. 2023;402(10402):656–66. 10.1016/S0140-6736(23)00918-2 37597892

[17] Miranda JJ, Diez-Canseco F, Araya R, Cutipe Y, Castillo H, Herrera V, et al. Transitioning mental health into primary care. Lancet Psychiatry. 2017;4(2):90–2. 28137383 10.1016/S2215-0366(16)30350-9 PMC8061534

[18] Toyama M, Castillo H, Galea JT, Brandt LR, Mendoza M, Herrera V, et al. Peruvian mental health reform: a framework for scaling-up mental health services. Int J Health Policy Manag. 2017;6(9):501–8. 28949462 10.15171/ijhpm.2017.07 PMC5582436

[19] MINSA. Salud mental comunitaria en el Peru: aportes temáticos para el trabajo con poblaciones. In: Ministerio de Salud, editor. Lima: Proyecto AMARES. 2006.

[20] Guerra M, Ferri CP, Sosa AL, Salas A, Gaona C, Gonzales V, et al. Late-life depression in Peru, Mexico and Venezuela: the 10/66 population-based study. Br J Psychiatry. 2009;195(6):510–5. 19949200 10.1192/bjp.bp.109.064055 PMC2915389

[21] World Health Organization. Global Health Observatory data repository: WHO; [updated 2019. Available from: https://apps.who.int/gho/data/node.main.MHHR?lang=en.

[22] Flores-Flores O, Zevallos-Morales A, Carrión I, Pawer D, Rey L, Checkley W, et al. “We can’t carry the weight of the whole world”: illness experiences among Peruvian older adults with symptoms of depression and anxiety. Int J Ment Heal Syst. 2020;14(1):49. 10.1186/s13033-020-00381-8 PMC7350592 32670400

[23] Flores-Flores O, Otero-Oyague D, Rey-Evangelista L, Zevallos-Morales A, Ramos-Bonilla G, Carrión I, et al. Agency and mental health among peruvian older adults during the COVID-19 lockdown. J Gerontol B Psychol Sci Soc Sci. 2023;78(6):1109–17. 36869737 10.1093/geronb/gbad040 PMC10214643

[24] CDC. Program to Encourage Active, Rewarding Lives [Available from: https://www.cdc.gov/prc/resources/tools/pearls.html#:~:text=PEARLS%20(Program%20to%20Encourage%20Active,all%2Dage%20adults%20with%20epilepsy.&text=More%20than%2050%20sites%20in,more%20organizations%20enrolling%20each%20year.

[25] Steinman LE, Gasca A, Hoeft TJ, Raue PJ, Henderson S, Perez R, et al. We are the sun for our community:" Partnering with community health workers/promotores to adapt, deliver and evaluate a home-based collaborative care model to improve equity in access to quality depression care for older U.S. Latino adults who are underserved. Front Public Health. 2023;11:1079319. 36817932 10.3389/fpubh.2023.1079319 PMC9932325

[26] Dimidjian S, Barrera M Jr, Martell C, Muñoz RF, Lewinsohn PM. The origins and current status of behavioral activation treatments for depression. Annu Rev Clin Psychol. 2011;7:1–38. 21275642 10.1146/annurev-clinpsy-032210-104535

[27] Nacional Instituto, de Estadistica e Informatica,. Proyecciones de Población Total según Departamento, Provincia y Distrito, 2018–2022. Lima: INEI; 2022.

[28] Instituto Nacional de Estadistica e Informatica. Encuesta Nacional de Hogares (ENAHO) 2022 Lima: INEI. 2022.

[29] Salud Md. Análisis Situacional de Salud Villa El Salvador. Lima. 2019.

[30] Dhalla S, Kopec JA. The CAGE questionnaire for alcohol misuse: a review of reliability and validity studies. Clin Invest Med. 2007;30(1):33–41. 17716538 10.25011/cim.v30i1.447

[31] Callahan CM, Unverzagt FW, Hui SL, Perkins AJ, Hendrie HC. Six-item screener to identify cognitive impairment among potential subjects for clinical research. Med Care. 2002;40(9):771–81. 12218768 10.1097/00005650-200209000-00007

[32] Marrone N, Ingram M, Bischoff K, Burgen E, Carvajal SC, Bell ML. Self-reported hearing difficulty and its association with general, cognitive, and psychosocial health in the state of Arizona, 2015. BMC Public Health. 2019;19(1):875. 31272444 10.1186/s12889-019-7175-5 PMC6609372

[33] Coyle CE, Steinman BA, Chen J. Visual acuity and self-reported vision status. J Aging Health. 2017;29(1):128–48. 26762396 10.1177/0898264315624909 PMC4940280

[34] Brooks R. EuroQol: the current state of play. Health Policy. 1996;37(1):53–72. 10158943 10.1016/0168-8510(96)00822-6

[35] Katz S. Assessing self-maintenance: activities of daily living, mobility, and instrumental activities of daily living. J Am Geriatr Soc. 1983;31(12):721–7. 6418786 10.1111/j.1532-5415.1983.tb03391.x

[36] Villagómez-Ornelas P, Hernández-López P, Carrasco-Enríquez B, Barrios-Sánchez K, Pérez-Escamilla R, Melgar-Quiñónez H. Validez estadística de la Escala Mexicana de seguridad alimentaria y la Escala Latinoamericana y Caribeña de seguridad alimentaria. salud pública de méxico. 2014;56:s5–11. 25649453 10.21149/spm.v56s1.5160

[37] Fried LP, Tangen CM, Walston J, Newman AB, Hirsch C, Gottdiener J, et al. Frailty in older adults: evidence for a phenotype. J Gerontol A Biol Sci Med Sci. 2001;56(3):M146–56. 11253156 10.1093/gerona/56.3.M146

[38] Moser A, Stuck AE, Silliman RA, Ganz PA, Clough-Gorr KM. The eight-item modified Medical Outcomes Study Social Support Survey: psychometric evaluation showed excellent performance. J Clin Epidemiol. 2012;65(10):1107–16. 22818947 10.1016/j.jclinepi.2012.04.007 PMC4119888

[39] Proctor E, Silmere H, Raghavan R, Hovmand P, Aarons G, Bunger A, et al. Outcomes for implementation research: conceptual distinctions, measurement challenges, and research agenda. Adm Policy Ment Health. 2011;38(2):65–76. 20957426 10.1007/s10488-010-0319-7 PMC3068522

[40] Proctor EK, Bunger AC, Lengnick-Hall R, Gerke DR, Martin JK, Phillips RJ, et al. Ten years of implementation outcomes research: a scoping review. Implement Sci. 2023;18(1):31. 37491242 10.1186/s13012-023-01286-z PMC10367273

[41] Weiner BJ, Lewis CC, Stanick C, Powell BJ, Dorsey CN, Clary AS, et al. Psychometric assessment of three newly developed implementation outcome measures. Implement Sci. 2017;12(1):108. 28851459 10.1186/s13012-017-0635-3 PMC5576104

[42] Aschbrenner KA, Kruse G, Gallo JJ, Plano Clark VL. Applying mixed methods to pilot feasibility studies to inform intervention trials. Pilot and Feasibility Studies. 2022;8(1):217. 36163045 10.1186/s40814-022-01178-x PMC9511762

[43] Farren L, Snowden M, Steinman L, Monroe-DeVita M. Development and Evaluation of a Fidelity Instrument for PEARLS. Front Public Health. 2015;2:200. 25964917 10.3389/fpubh.2014.00200 PMC4410416

[44] Villarreal-Zegarra D, Copez-Lonzoy A, Bernabé-Ortiz A, Melendez-Torres GJ, Bazo-Alvarez JC. Valid group comparisons can be made with the Patient Health Questionnaire (PHQ-9): A measurement invariance study across groups by demographic characteristics. PLoS ONE. 2019;14(9):e0221717. 31498796 10.1371/journal.pone.0221717 PMC6733536

[45] Calderón M, Gálvez-Buccollini JA, Cueva G, Bromley C, Fiestas F. Validación de la versión peruana del PHQ-9 para el diagnóstico de depresión. evista Peruana de Medicina Experimental y Salud Publica. 2012;29:578. 10.1590/S1726-46342012000400027 23338650

[46] Spitzer RL, Kroenke K, Williams JB, Löwe B. A brief measure for assessing generalized anxiety disorder: the GAD-7. Arch Intern Med. 2006;166(10):1092–7. 16717171 10.1001/archinte.166.10.1092

[47] Villarreal-Zegarra D, Barrera-Begazo J, Otazú-Alfaro S, Mayo-Puchoc N, Bazo-Alvarez JC, Huarcaya-Victoria J. Sensitivity and specificity of the Patient Health Questionnaire (PHQ-9, PHQ-8, PHQ-2) and General Anxiety Disorder scale (GAD-7, GAD-2) for depression and anxiety diagnosis: a cross-sectional study in a Peruvian hospital population. BMJ Open. 2023;13(9):e076193. 37714674 10.1136/bmjopen-2023-076193 PMC10510859

[48] Pedroso-Chaparro MdS, Márquez-González M, Fernandes-Pires J-A, Gallego-Alberto L, Jiménez-Gonzalo L, Nuevo R, et al. Validation of the Spanish version of the Three-Item Loneliness Scale (Validación de la versión española de la Escala de Soledad de Tres Ítems). Studies in Psychology. 2022;43(2):311–31.

[49] Trucharte A, Calderón L, Cerezo E, Contreras A, Peinado V, Valiente C. Three-item loneliness scale: psychometric properties and normative data of the Spanish version. Curr Psychol. 2023;42(9):7466–74. 34305365 10.1007/s12144-021-02110-x PMC8285042

[50] Moher D, Hopewell S, Schulz KF, Montori V, Gøtzsche PC, Devereaux PJ, et al. CONSORT 2010 explanation and elaboration: updated guidelines for reporting parallel group randomised trials. BMJ. 2010;340:c869. 20332511 10.1136/bmj.c869 PMC2844943

[51] Guetterman TC, Fetters MD, Creswell JW. Integrating quantitative and qualitative results in health science mixed methods research through joint displays. Ann Fam Med. 2015;13(6):554–61. 26553895 10.1370/afm.1865 PMC4639381

[52] Boeije H. A purposeful approach to the constant comparative method in the analysis of qualitative interviews. Qual Quant. 2002;36(4):391–409. 10.1023/A:1020909529486

[53] Ciechanowski P, Wagner E, Schmaling K, Schwartz S, Williams B, Diehr P, et al. Community-integrated home-based depression treatment in older adults: a randomized controlled trial. JAMA. 2004;291(13):1569–77. 15069044 10.1001/jama.291.13.1569

[54] Smith ML, Steinman LE, Montoya CN, Thompson M, Zhong L, Merianos AL. Effectiveness of the program to encourage active, rewarding lives (PEARLS) to reduce depression: a multi-state evaluation. Front Public Health. 2023;11:1169257. 37361168 10.3389/fpubh.2023.1169257 PMC10289834

[55] Bowling CB, Thomas J, Gierisch JM, Bosworth HB, Plantinga L. Research inclusion across the lifespan: a good start, but there is more work to be done. J Gen Intern Med. 2023;38(8):1966–9. 37002458 10.1007/s11606-023-08182-8 PMC10272002

[56] Loper A, Woo B, Metz A. Equity is fundamental to implementation science. Stanf Soc Innov Rev. 2021;19(3):A3–5.

[57] Vetrano DL, Calderón-Larrañaga A, Marengoni A, Onder G, Bauer JM, Cesari M, et al. An international perspective on chronic multimorbidity: approaching the elephant in the room. J Gerontol A Biol Sci Med Sci. 2018;73(10):1350–6. 28957993 10.1093/gerona/glx178 PMC6132114

[58] Pan-American Health Organization. Noncommunicable diseases and mental health Washington DC 2023 [Available from: https://www.paho.org/en/noncommunicable-diseases-and-mental-health.

